# Some examples of privacy-preserving sharing of COVID-19 pandemic data with statistical utility evaluation

**DOI:** 10.1186/s12874-023-01927-3

**Published:** 2023-05-19

**Authors:** Fang Liu, Dong Wang, Tian Yan

**Affiliations:** 1 Department of Applied and Computational Mathematics and Statistics, University of Notre Dame, Notre Dame, 46556 IN USA; 2 College of Cyberspace Security, Hangzhou Dianzi University, Wuhan, 430079 China

**Keywords:** COVID-19 pandemic, Differential privacy, Geo-indistinguishability, Hotspot heat maps, Contact tracing network, Synthetic data

## Abstract

**Background:**

A considerable amount of various types of data have been collected during the COVID-19 pandemic, the analysis and understanding of which have been indispensable for curbing the spread of the disease. As the pandemic moves to an endemic state, the data collected during the pandemic will continue to be rich sources for further studying and understanding the impacts of the pandemic on various aspects of our society. On the other hand, naïve release and sharing of the information can be associated with serious privacy concerns.

**Methods:**

We use three common but distinct data types collected during the pandemic (case surveillance tabular data, case location data, and contact tracing networks) to illustrate the publication and sharing of granular information and individual-level pandemic data in a privacy-preserving manner. We leverage and build upon the concept of differential privacy to generate and release privacy-preserving data for each data type. We investigate the inferential utility of privacy-preserving information through simulation studies at different levels of privacy guarantees and demonstrate the approaches in real-life data. All the approaches employed in the study are straightforward to apply.

**Results:**

The empirical studies in all three data cases suggest that privacy-preserving results based on the differentially privately sanitized data can be similar to the original results at a reasonably small privacy loss ($\epsilon \approx 1$). Statistical inferences based on sanitized data using the multiple synthesis technique also appear valid, with nominal coverage of 95% confidence intervals when there is no noticeable bias in point estimation. When $\epsilon <1$ and the sample size is not large enough, some privacy-preserving results are subject to bias, partially due to the bounding applied to sanitized data as a post-processing step to satisfy practical data constraints.

**Conclusions:**

Our study generates statistical evidence on the practical feasibility of sharing pandemic data with privacy guarantees and on how to balance the statistical utility of released information during this process.

**Supplementary Information:**

The online version contains supplementary material available at 10.1186/s12874-023-01927-3.

## Introduction

### Background

A huge amount of data of various types have been collected during the COVID-19 pandemic, the analysis and interpretation of which have been indispensable to health authorities and experts to gain an understanding of the disease, identify risk factors, monitor and forecast the spread of the disease, to evaluate the impacts of the pandemic on different aspects of our society, and to implement strategies that mitigate negative impacts. As the pandemic shifts to an endemic state, the collected data will continue to serve as rich sources for further research on the disease and its impacts to prepare us for future pandemics.

Naïve release and sharing of the pandemic data can be associated with serious privacy concerns, especially considering that a huge amount and a great variety of data were collected quickly in a short period of time and the data privacy and ethics regulations were lagging behind at least in the initial stage of the pandemic. Many types of collected data are known to be associated with high privacy risk, such as disease status, medical history, locations, close contacts, employment/income status, etc. Privacy protection must be considered when sharing and releasing data collected during the pandemic. Fortunately, this is not an unsolvable problem. Research questions of interest often revolve around learning population-level and aggregate information while privacy attacks focus on learning individual-level information. Therefore, if a privacy-preserving procedure can maintain accurate and useful aggregate information while guaranteeing individual-level privacy, it would make a potentially effective approach for data sharing.

### Related work

Various types of privacy-preserving collection and analysis of COVID-19 data were conducted during the pandemic. Google research teams applied differential privacy (DP) to generate anonymized metrics from the data of Google users who opted in for the Location History setting in their Google accounts and produce the COVID-19 community mobility reports [2], to understand the impacts of social distancing policies on mobility and COVID-19 case growth in the US [57], to generate anonymized trends in Google searches for COVID-19 symptoms and related topics [23], and to forecast COVID-19 trends using spatiotemporal graph neural networks [33]. DP was integrated into deep learning to predict COVID-19 infections from imaging data [46, 54]. Butler et al. [12] applied DP to generate individual-level health tokens/randomized health certificates while allowing useful aggregate risk estimates to be calculated.

There also exist privacy-preserving technologies and tools that protect sensitive information in location data and proximity data. These types of data were instrumental to track the trajectory of a COVID-19 case and for contact tracing (CT) so to identify people who might have close contact with COVID-19 patients. On the other hand, location and relational information can be highly revealing of personal information in general. Privacy-preserving technologies and tools were developed and adopted in CT apps and software around the world during the pandemic to track the spread of the disease. The apps collect users’ location data (e.g., GPS) or proximity data (e.g., Bluetooth), via either a centralized (e.g., Alipay Health Code and WeChat in China [28], Corona100m in South Korea [58], COVIDTracker in Thailand [1], ProteGo in Poland [25], and Pan-European Privacy-Preserving Proximity Tracing (PEPP-PT) in EU [49]) or decentralized model (Safe paths [50] and the proximity-based Google/Apple Exposure Notification (GAEN) system [4] in the US) to identify and notify those who might have been near a COVID-19 patient and at high risk of contracting the disease. We refer readers to Wang and Liu [56] for a comprehensive review of the CT apps used during the pandemic.

### Our work and contributions

Many privacy-preserving methods developed and implemented during the pandemic, including the work mentioned in Section “Related Work”, focus on information shared with governments, health officials, and the public so to facilitate quick decision-making and timely actions during the pandemic. In contrast, privacy-preserving COVID-19 data release for research use has received less attention, which is the major focus of our work. Sharing data for research use is not only critical for making scientific discoveries, but also for producing real-world evidence and generating new insights into how we can better handle similar crises in the future. Data for research use often contain more granular information compared to those shared with decision-makers and the public and are thus associated with higher privacy risks that must be mitigated before release, the topic we address in this work. We focus on the privacy-preserving release of synthetic data generated at a pre-specified privacy budget. With synthetic data, data users may perform analysis on their own [8]. In summary,

- We leverage and build upon existing DP concepts and techniques and apply them to several common but distinct pandemic data types – surveillance data, case location data, and Contact Tracing Networks (CTNs) to demonstrate the publication of pandemic data with formal privacy guarantees. These three data types were routinely collected during the pandemic, provide different information on COVID-19, are distinct in terms of data structure and statistical analysis, and are all subject to privacy risks.
- For case surveillance data, we use the flat Laplace sanitizer with DP guarantees and examine the statistical utility of log-linear models based on sanitized data in simulated data and real data published by the U.S. CDC. Our results suggest that simple approaches such as the flat Laplace sanitizer can be effective for releasing granular case surveillance data, providing a good balance between privacy and data utility.
- For location data, we demonstrate the application of the planar Laplace mechanism with geo-indistinguishability guarantees to simulation data and a real South Korean case location dataset to examine inference from cluster point process models and the accuracy of hotspot heat maps based on sanitized locations. The method would be particularly useful for protecting location privacy when sharing information at a local level or releasing hotspot heat maps on a relatively fine scale.
- For CTNs, we apply DP exponential random graph model (ERGM) to generate privacy-preserving synthetic networks and investigate the utility of sanitized networks in inference from ERGMs and the preservation of descriptive structural network statistics. The results suggest DP-ERGM is relatively insensitive to $\epsilon$ and implies that small $\epsilon$ can be used to provide strong privacy guarantees without sacrificing much of the utility.
- Our study generates statistical evidence on the practical feasibility of sharing different types of pandemic data with formal privacy guarantees. The approaches examined in this study do not target learning individual-level information but focus on preserving aggregated and population-level information.

The rest of the paper is organized as follows. Section “Preliminaries” provides an overview of the basic concepts in DP, some common randomized mechanisms for achieving DP, and an approach for obtaining valid inferences from sanitized data. Sections “Privacy-preserving case surveillance data release”, “Privacy-preserving release of case location data” and “Privacy-preserving sharing of contact tracing networks” apply DP procedures to release privacy-preserving case surveillance data, case location data, and CTNs, respectively, conduct simulation studies to examine the statistical utility of the privacy-preserving data, and apply the DP procedures to real pandemic data. Section “Conclusions” provides some final remarks on the implementations of DP methods in releasing COVID-19 data.

## Preliminaries

We provide a brief overview of some common DP concepts and mechanisms. The overview does not aim at covering every concept in DP but rather focuses on those used or mentioned in this paper.

### Differential privacy

#### Definition 1

($(\epsilon ,\delta )$-DP [17, 19]). A randomized algorithm $\mathcal {M}$ is of $(\epsilon ,\delta )$-DP if for all dataset pairs of neighboring data sets $(D,D^{\prime })$ differing by one record and for all subsets $\mathcal {S}\subseteq$ image$(\mathcal {M})$,

1 $$\begin{aligned} \textrm{Pr}(\mathcal {M}(D)\in \mathcal {S}) \le e^{\epsilon } \textrm{Pr}(\mathcal {M}(D^{\prime })\in \mathcal {S})+\delta . \end{aligned}$$

*D* and $D^{\prime }$ differing by one record (denoted by $d(D,D^{\prime })=1$) may refer to the case that they are of the same size but differ in at least one attribute value in exactly one record (bounded DP), or $D^{\prime }$ has one record less than *D* or vice versa (unbounded DP) [35]. $\epsilon >0$ and $\delta \ge 0$ are privacy budget or privacy loss parameters. When $\delta =0$, $(\epsilon ,\delta )$-DP becomes pure $\epsilon$-DP; the smaller $\epsilon$ is, the more privacy protection there is on any individual in the data, as the released results $\mathcal {M}(D)$ and $\mathcal {M}(D^{\prime })$ are similar in the sense that their probability density/mass function ratio is bounded with $(e^{-\epsilon }, e^{\epsilon })$. There is no consensus and lacks a universal guideline on the choice of $\epsilon$ [18]. $\epsilon$ typically ranges from $10^{-3}$ to 10 in empirical studies in the DP literature, depending on the type of information released, social perception of privacy, and expected accuracy of released data, among others. Real-life applications of DP often employ larger $\epsilon$ for better utility (e.g., US Census uses $\epsilon$ of 19.61 [11] and Apple Inc. sets $\epsilon$ at 2, 4, or 8 for different Apps [5]). $\delta$, if not 0, is often set at a very small value (inversely proportional to poly(*n*)) and can be interpreted as the probability that the pure $\epsilon$-DP is violated.

Definition 1 is the original DP definition. Relaxed versions and extensions exist, such as $(\epsilon ,\delta )$-probabilistic DP (pDP) [43], $(\epsilon ,\tau )$-concentrated DP (CDP) [20], zero-concentrated DP [10] (zCDP), Rényi DP (RDP) [45], and Gaussian DP (GDP) [14].

DP provides a mathematically rigorous framework for protecting individual privacy when releasing and sharing information. Many mechanisms and procedures have been developed to achieve DP. In this paper, we employ the Laplace mechanism with pure $\epsilon$-DP to illustrate how to apply DP concepts and procedures to protect individual privacy when releasing COVID-19 data. When other types of DP guarantees are desired, such as $(\epsilon ,\delta )$-(p)DP, corresponding mechanisms can be used, such as the Gaussian mechanism [16, 38].

#### Definition 2

(Laplace mechanism [19]). Let $\textbf{s}=(s_1,\ldots ,s_r)$ be a statistic calculated from a dataset. The *Laplace mechanism* of $\epsilon$-DP releases $\textbf{s}^{*}=\textbf{s}+\textbf{e}$, where $\textbf{e}$ contains *r* independent samples from Laplace$\left( 0,\Delta \epsilon ^{-1}\right)$, where $\Delta _1={max}_{{x,x', d(x,x')=1}} \Vert \textbf{s}(x)-\textbf{s}(x')\Vert _1$ is the $\ell _1$ *global sensitivity* of $\textbf{s}$.

The $\ell _1$ global sensitivity represents the maximum $\ell _1$ change in $\textbf{s}$ between two neighboring data sets (in general, one can define $\ell _p (p\ge 0)$ global sensitivity; see [38]). The larger the sensitivity, the more impact a single individual has on the value of $\textbf{s}$, and more noise would be needed to achieve $\epsilon$-DP.

Every time a dataset is queried, there is a privacy cost (loss) on the individuals in the dataset. Data curators need to track the privacy cost during the querying process to ensure the overall privacy spending does not exceed a pre-specified level. Two basic composition principles in DP, *parallel composition* and *sequential composition* [44], can be used in privacy loss accounting, which are also used in later sections of the paper.

#### Definition 3

(Basic privacy loss composition of $(\epsilon ,\delta )$-DP [44]). If mechanism $\mathcal {M}_j$ of $(\epsilon _j,\delta _j)$-DP is applied to disjoint dataset $D_j$ for $j=1,\dots ,P$, the parallel composition states the total privacy loss in data $\cup _j{D_j}$ from apply the *P* mechanisms $\mathcal {M}_j$ for $j=1,\dots ,P$ is $(\max \{\epsilon _j\}, \max \{\delta _j\})$; if $\mathcal {M}_j$ is applied to the same dataset *D*, the sequential composition states that the total privacy loss in *D* is $(\sum _j\epsilon _j, \sum _j\delta _j)$ from applying the *P* mechanisms $\mathcal {M}_j$ for $j=1,\dots ,P$.

In layman’s terms, the two privacy loss composition principle states as long as there is no overlapping information between two datasets to which two DP mechanisms are applied, the overall loss for releasing the query results is the maximum privacy spending between the two; otherwise, the loss adds up. The sequential composition on $(\epsilon ,\delta )$-DP can be over-conservative for repeated queries on the same data; advanced composition [21] for $(\epsilon ,\delta )$-DP and the relaxed DP notions mentioned above (e.g., CDP, zCDP, RDP, GDP) all achieve tighter total privacy loss bound than the basic composition.

DP is a mainstream concept in privacy research and applications nowadays. Backed up by its mathematical rigor and robustness to various privacy attacks, the properties it has, including privacy loss composition, immunity to post-processing, and being future-proof, make it attractive for designing sophisticated DP procedures and algorithms for complicated analysis and learning problems. Immunity to post-processing and being future-proof refer to instances that information released from a DP mechanism won’t leak additional information about the individuals in the dataset on which the information is based when it is further processed after the release or when there is additional information on these individuals in the future from other sources, as long as the original data is not accessed.

### Geo-indistinguishability

Andrés et al. [3] extend the pure $\epsilon$-DP concept to releasing privacy-preserving location data that are represented as pairs of 2-dimensional GPS coordinates, along with the planar Laplace mechanism to achieve such privacy guarantees.

#### Definition 4

(Geo-indistinguishability (GI) [3]). Let $d(P,P^{\prime })$ denote the Euclidean distance between any two distinct locations *P* and $P^{\prime }$, and $\epsilon$ be the unit-distance privacy loss. A randomized mechanism $\mathcal {M}$ satisfies $\epsilon$-GI if and only, for any $\gamma >0$, any possible released location $P^{*}$, and all possible pairs of *P* and $P^{\prime }$ that $d(P,P^{\prime })\le \gamma$,

2 $$\begin{aligned} \textrm{Pr}(\mathcal {M}(P)=P^{*}|P)\le e^{\epsilon \gamma }\cdot \textrm{Pr}(\mathcal {M}(P^{\prime })=P^{*}|P^{\prime }). \end{aligned}$$

$\mathcal {M}$ in Eq. (2) enjoys $(\epsilon \gamma )$-GI for any specified $\gamma >0$ in the sense that the probability of distinguishing any two locations within a radius of $\gamma$, given the released location $P^{*}$, is $e^{\epsilon \gamma }$-fold the probability when not having $P^{*}$. $\epsilon$ is the per-unit-distance loss and $\gamma$ denotes how many units. The larger $\epsilon$ is, the larger the privacy loss $(\epsilon \gamma )$ is and the higher probability of identifying the true location information within a radius of $\gamma$ mile given the perturbed location information. Though increasing $\gamma$ would also lead to higher privacy loss and the probability of identifying the true location is within a radius of $\gamma$ but the large $\gamma$ would make this identification less meaningful.

#### Definition 5

(planar Laplace mechanism [3]). Let the coordinates of the observed location *P* in the Euclidean space by (*x*, *y*). The planar Laplace mechanism of $\epsilon$-GI generates sanitized location $P^{*}$ with coordinates

3 $$\begin{aligned}{} & {} (x^{*},y^{*}) =(x+r\cos (\theta ), y+r\sin (\theta )), { where}\end{aligned}$$

4 $$\begin{aligned}{} & {} r \sim {gamma }(2,\epsilon )=r\epsilon ^2 e^{-\epsilon r}\\{} & {} \theta \sim {uniform }(0,2\pi )=1/(2\pi ). \end{aligned}$$

*r* in Eq. (3) is the distance between $P^{*}$ and *P* and $\theta$ is the angle of $P\rightarrow P^{*}$ in the Euclidean space, and *r* and $\theta$ are independent. The concepts of GI and planar Laplace mechanism are employed in Section “Privacy-preserving release of case location data” for releasing privacy-preserving location data.

Precisely speaking, GI is more related to local DP [15], an extension of the pure $\epsilon$-DP, than the latter per se, which is often used for releasing aggregate information rather than an individual response.

#### Definition 6

($\epsilon$-local DP [15]). A randomization mechanism $\mathcal {M}$ provides $\epsilon$ local DP if $\textrm{Pr}[\mathcal {M}(x)\in \Omega ]\le {e^{\epsilon }}\cdot \textrm{Pr}[\mathcal {M}(x')\in {\Omega }]$ for all pairs of possible data points *x* and $x'$ from an individual and all possible output subset $\Omega$ from $\mathcal {M}$.

### Privacy-preserving statistical inference

Sanitized outputs, compared to the original outputs, are subject to an extra source of variability due to the noise introduced through the randomized algorithm $\mathcal {R}$ for achieving DP. To account for the extra source of variability for valid statistical inference, one may directly model the sanitization mechanism, which may complicate the regular inferential procedures either analytically or computationally and is problem-specific. An alternative is the multiple syntheses (MS) approach that releases multiple sets of sanitized datasets or statistics and employs an inferential rule across the multiple sets to obtain valid inference [40]. The MS approach is general and straightforward to apply. We adopt the MS approach to obtain privacy-preserving inference from sanitized data in this paper.

Denote the number of released sets by *m*. Per sequential composition, the total privacy budget would be split into *m* portions, one per release. $m\in [3,5]$ is recommended [40]. WLOS, suppose the parameter of interest is $\beta$ and its *l*-th sanitized estimate is $\hat{\beta }^{(l)}$ with estimated variance $w^{(l)}$ for $l=1,\ldots ,m$. The final inference of $\beta$, including hypothesis testing and confidence interval (CI) construction, is based on the following inferential rule.

5 $$\begin{aligned}{} & {} \bar{\beta }=m^{-1}{\sum }_{l=1}^m\hat{\beta }^{(l)},\; T=m^{-1}B+W \end{aligned}$$

6 $$\begin{aligned}{} & {} (\beta -\bar{\beta })T^{-1/2}\sim t_{\nu =(m-1)(1+mW/B)^2}, \textrm{ where} \\{} & {} B={\sum }_{l=1}^m(\hat{\beta }^{(l)}-\bar{\beta })^2/(m-1)\ \text {(between-set variability)} \nonumber \\{} & {} W=m^{-1}{\sum }_{l=1}^m w^{(l)}\ \text {(within-set variability).} \nonumber \end{aligned}$$

### Overview of case surveillance data, case location data, and contact tracing networks (CTNs)

We present the privacy-preserving release of three pandemic data types: subgroup case surveillance data (Section “Privacy-preserving case surveillance data release”), case location data (Section “Privacy-preserving release of case location data”), and CTNs (Section “Privacy-preserving sharing of contact tracing networks”). In each case, we describe data characteristics, introduce methods for sanitization, conduct a simulation study to examine the impact of sanitization on statistical inference, and apply the method to a real data set when one is available. We choose the three data types because they were routinely collected during the pandemic, are distinct in terms of data structure and statistical analysis, and provide different information on COVID-19.

Case surveillance data are a listing of cases, together with attributes associated with the cases, such as demographics, exposure histories, etc. Surveillance data are crucial during the pandemic for monitoring and forecasting the spread of the disease, understanding how COVID impacts the capacity of healthcare systems and providing necessary information to health authorities for quick decision-making. Case numbers reported at different geographical scales by demographic groups such as age, gender, race, and ethnicity provide valuable information for identifying risk factors and groups vulnerable to the disease and understanding the heterogeneity of the susceptibility to the disease. On the other hand, publishing such granular information may lead to re-identification and disclosure risk, especially when data are sparse. This section focuses on publishing granular case numbers with privacy guarantees.

Location history data may be collected by health authorities when a person is diagnosed with COVID-19 and interviewed about his or her whereabouts in the past few weeks [13, 48]. Patient location data are critical for health authorities to take measures to limit the spread of the disease. With individual-level location data, researchers can conduct spatial data analysis such as using point process models to understand the spatial trend of the cases or generating COVID-19 hotspot heat maps. However, location information, if shared as is, may cause serious privacy risks for the patients and can even lead to cyber-bullying [47].

Contact tracing (CT) is an effective approach for curbing the spread of COVID-19 during the pandemic. CT can be carried out manually by human tracers or digitally via GPS or Bluetooth devices. CT networks (CTNs), constructed from CT data, can be regarded as a social network, where individuals are the nodes and an edge between two people represents close contact between them (e.g., within 6 feet of each other for a cumulative total of 15 minutes or more over a 24-hour period). CTNs are of research interest as they provide information to better understand how physical proximity affects the spread of the disease and human contact behaviors during the pandemic, among others. However, sharing CTNs as is has privacy concerns as adversaries may link a CTN with other databases or use background knowledge to infer who was infected with COVID-19 and tell who was close physically (appearing in the same place at the same time) based on the edge information in a CTN.

In summary, surveillance data help better understand risk factors associated with COVID-19 and identify sub-populations that are vulnerable to the disease; location data can be used to explore relationships between hotspots and residential characteristics to study issues such as residential racism and structural segregation during the pandemic, CTNs allow us to study how clustering of COVID-19 cases and how physical proximity may affect the spread of the disease, among others. Meanwhile, all three data types contain sensitive information and are subject to privacy risks, and may not be shared without privacy protection considerations.

## Privacy-preserving case surveillance data release

An example of case surveillance data is the COVID-19 death count data released by the U.S. CDC website. Table 1 shows such a dataset we downloaded on May 24, 2022 (Table 2 at https://www.cdc.gov/nchs/nvss/vsrr/covid19/health_disparities.htm) with some minor modifications (we removed the race group “unknown’ and collapsed age groups (0, 4] and [5, 17] to a single $<\!18$ group, and age groups [75, 84] and $\ge \!85$ to a single $>\!74$ group). Table 1 contains two attributes – age group and race/ethnicity; each has 7 levels, leading to a $7\times 7$ contingency table. The sample size is $n\!=\!998,262$, assumed to be public information.

**Table 1:** U.S. COVID-19 death counts by age and race/ethnicity (May 24, 2022)

Age (ys)	Race/Ethnicity							
group	NH White	NH Black	NH AIAN	NH Asian	NH NHPI	NH Mix	Hispanic	Total
<17	387	274	15	36	11	30	303	1056
18-29	2263	1492	187	190	49	73	2015	6269
30-39	6661	4144	560	558	151	157	5919	18150
40-49	17269	8937	1021	1206	265	309	13981	42988
50-64	97418	35753	3198	5312	715	952	43657	187005
65-74	141409	37765	2901	7423	501	913	38422	229334
>75	380630	54576	3210	16504	449	1380	56711	513460
Total	646037	142941	11092	31229	2141	3814	161008	998262

Race/ethnicity = ‘unknown’ is not included in the table

*NH *Non-Hispanic, *AIAN *American Indian or Alaska Native, *NHPI *Native Hawaiian or Other Pacific Islander, “*Mix*” means “more than one race”

### Method

Publishing a privacy-preserving case number dataset can be formulated as releasing a multi-dimensional histogram or contingency table. The most straightforward approach for achieving DP when releasing a histogram and contingency table is the flat Laplace sanitizer, which injects noise from the Laplace mechanism directly into each cell count in a histogram or contingency table; methods that achieve better utility in sanitizing count data for certain analyses exist, at the cost of more complicated implementation, such as [9, 22, 26, 30, 37, 59–61, 63], just to name a few. Given that there exist many methods for sanitizing count data, many aiming at improving the utility of a certain type of analysis and not straightforward to implement, and our main goal is to demonstrate the application of DP in releasing count data in general without a specific downstream analysis task in mind, we employ the flat Laplace mechanism (we examined a couple of other approaches, but their performance is not as good as Laplace sanitizer in the in utility analysis. More details are provided in Section “Summary”).

In our problem setting, the Laplace sanitizer employs the Laplace mechanism in Definition 2 to sanitize each cell count of the multidimensional histogram/ contingency table to be released. The $l_1$ global sensitivity of releasing a histogram/table is 1 (WLOS, we use the unbounded DP unless mentioned otherwise; the sensitivity is 2 if the bounded DP is used). Sanitized count in cell *k* is $\tilde{y}_k \sim$ Laplace$(y_k,\epsilon ^{-1})$ for $k=1,\ldots ,K$ cells. Sanitized counts may be negative as the support of the Laplace distribution is the real line. There are two ways to deal with this problem – to replace negative values with 0 and to re-draw until the sanitized value is non-negative [39]. In either case, normalization would be needed if the total sample size *n* is fixed. Real non-negative sanitized counts can be rounded to obtain integer counts without compromising privacy due to the immunity to post-processing property. Algorithm 1 lists the steps of the procedure.

**Figure:**
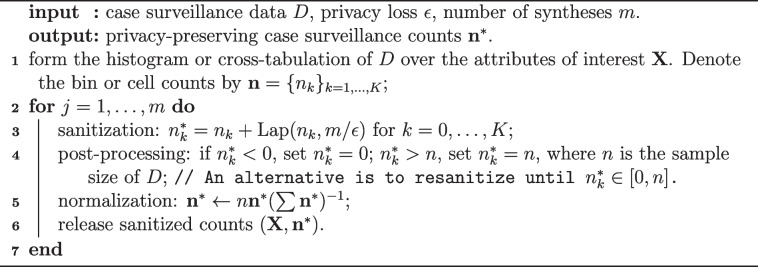
**Algorithm 1** Privacy-preserving release of case surveillance data via flat Laplace sanitizer

To obtain sanitized counts for a lower-dimensional histogram/contingency table from the sanitized histogram/table at a more granular level, one may sum sanitized counts over corresponding cells to obtain cell counts in the lower-dimensional histogram/table. Per the immunity to post-processing property, the summed counts are also privacy-preserving but are subject to a larger sanitization variability since each contains the sum of multiple independent noise terms.

### Simulation study

We use a simulation study to study how DP sanitization affects statistical inference based on sanitized count data. We simulated 1,000 datasets from $y_k\sim$ multinomial($n, p_k$), where $p_k=\lambda _k/(1+\lambda _k)$, $\log (\lambda _k)=\beta _0+\beta _1 x_{k1}+\beta _2 x_{k2} + \beta _3 x_{k3}+ \beta _4 x_{k1}x_{k2} + \beta _5 x_{k1}x_{k3} + \beta _6 x_{k2}x_{k3}$ for $k=1,\ldots ,8$ and $X_1=\{0,1\}, X_2=\{0,1\}, X_3=\{0,1\}$ are binary attributes. In each dataset, we sanitize $\textbf{y}=\{y\}_{k=1,\ldots ,8}$ via the flat Laplace sanitizer independently for $m=3$ times to obtain differentially private $\tilde{\textbf{y}}^{(l)}$ and $l=1,\ldots ,m$, each at a privacy budget of $\epsilon /m$, where $\epsilon$ is the total privacy budget. We examine two sample sizes at $n=200$ and $n=1,000$ and four privacy loss parameters at $\epsilon =0.5,1,2$ and 5. We assume the total sample size *n* is fixed and normalize the raw sanitized counts from the flat sanitizer via $n\tilde{y}_k^{(l)}/\sum _l\tilde{y}_k^{(l)}$. For utility check, we run the loglinear model $\log (\lambda _k)=\beta _0+\beta _1 x_{k1}+\beta _2 x_{k2} + \beta _3 x_{k3}+ \beta _4 x_{k1}x_{k2} + \beta _5 x_{k1}x_{k3} + \beta _6 x_{k2}x_{k3}$ for $k=1,\ldots ,8$, assuming $\tilde{\textbf{y}}^{(l)}_k\!\sim$ Poisson($\lambda _k$), on each set of sanitized data to obtain inference on $\beta _1,\ldots ,\beta _6$ using Eqs. (5) and (6). For comparison, we also run the same log-linear model on the original $\textbf{y}$.

**Fig. 1:**
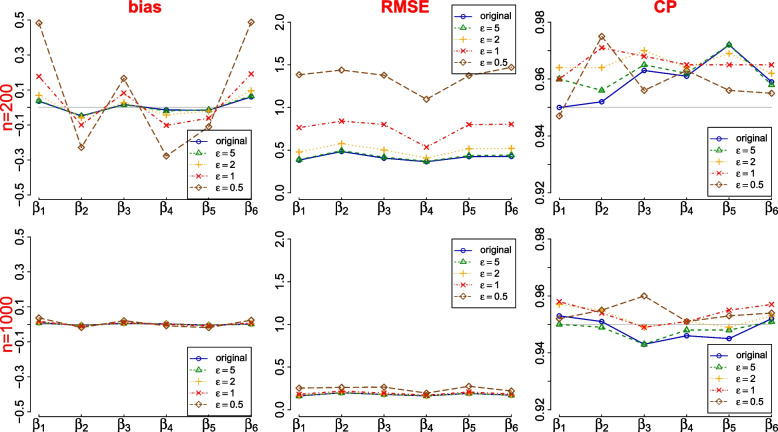
Privacy-preserving inference in the log-linear model on sanitized counts obtained via the flat Laplace sanitizer in simulated data (1000 repeats; $m=3$)

The results are presented in Fig. 1 and the main observations are summarized as follows. The smaller $\epsilon$ or *n* is, the more impact the DP procedure has on the inference; i.e., larger bias and larger root mean squared error (RMSE). Regardless of *n* or $\epsilon$, the coverage probability (CP) of the 95% CIs is always at the nominal level. At $n=1,000$, the inference is barely affected by the DP sanitization even for $\epsilon =0.5$. At $n=200$, the bias is noticeable with relatively large RMSE for $\epsilon =0.5$, acceptable at $\epsilon =1$, and almost ignoble for $\epsilon >1$, compared to the original inference.

### Application to CDC case surveillance data

We apply the flat Laplace sanitized to the CDC in Table 1. If released data are not used for statistical inference or uncertainty quantification, we may release a single sanitized tabular dataset ($m=1$). Let $\tilde{y}_k=y_k+e_k$, where $e_k\sim$ Laplace($0,\epsilon ^{-1}$), for $k=1,\ldots ,49$ independently. Since $n=998,262$ is public knowledge, the sanitized $\tilde{y}_k$ is normalized as in $\tilde{y}_k\leftarrow n\tilde{y}_k/\sum _k{\tilde{y}_k}$ to keep the total *n* at 998, 262. An example sanitized dataset at $\epsilon =0.5$ is given in Table 2. There is some fluctuation in each cell count due to the sanitization, as expected. The column and row marginals are calculated by summing over the corresponding cell counts after sanitization.

**Table 2:** Flat Laplace sanitized ($\epsilon =0.5, m=1$) US COVID-19 death counts by age group and race/ethnicity on May 24, 2022

Age (ys)	Race/Ethnicity							
group	NH White	NH Black	NH AIAN	NH Asian	NH NHPI	NH Mix	Hispanic	Total
<17	385	271	14	37	8	29	308	1052
18-29	2258	1491	186	198	49	72	2009	6263
30-39	6664	4140	562	558	145	156	5928	18153
40-49	17269	8937	1021	1202	266	299	13982	42976
50-64	97421	35753	3195	5311	713	952	43658	187003
65-74	141413	37766	2897	7427	501	914	38425	229343
>75	380642	54577	3209	16505	449	1379	56712	513472
Total	646053	142935	11084	31238	2130	3801	161021	998262

Race/ethnicity = ’unknown’ is not included in the table

* NH* Non-Hispanic, *AIAN *American Indian or Alaska Native, *NHPI *Native Hawaiian or Other Pacific Islander, “*Mix*” means “more than one race”

**Fig. 2:**
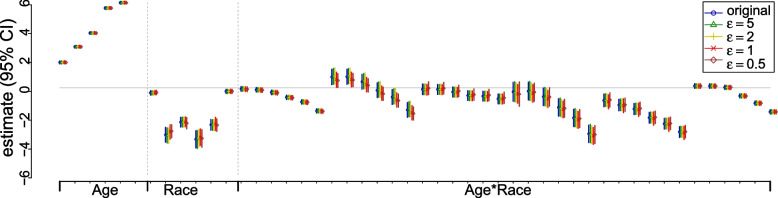
Privacy-preserving results from the log-linear model on sanitized CDC COVID-19 death data via the flat Laplace sanitizer

If released data is used for statistical inference, we can use the MS approach to release multiple sets of sanitized tables. We sanitized $y_k$ with noise from Laplace($0,\epsilon /m)$ independently to obtain $m=3$ sets of sanitized $\tilde{y}_k^{(l)}$ for $l=1,2,3$. Some examples of sanitized data are provided in the supplementary materials. For the statistical analysis on the sanitized data, we fitted a 2-way log-linear model with covariates age group and race/ethnicity (other analyses can also be run, such as logistic regression and Chi-squared test). There are 48 regression coefficients – 6 associated with age ($<18$ years is the reference group), 6 associated with race (non-Hispanic white is the reference group), and 36 parameters representing the interaction between the two. The estimates of the regression coefficients are presented in Fig. 2. In summary, the privacy-preserving inferences based on the sanitized counts are similar to the original inference at all $\epsilon$ values, largely due to the large sample size of the data.

### Summary

Case number data with granular information permits more complicated analysis and helps us understand better the pandemic, such as quantifying the effects of risk factors for COVID-19 as demonstrated in Fig. 2). We demonstrate via a simulation study and a real data application that useful privacy-preserving can be achieved, especially when *n* is large or people are willing to sacrifice some privacy ($\epsilon$ is not too small). The results also suggest the flat Laplace sanitizer can be an effective approach for that purpose, despite its simplicity.

Though we focus on the flat Laplace sanitizer for demonstration purposes, we also run a couple of other methods that sanitize count data in a hierarchical manner in the simulation study and the case study. The two approaches are – - the universal histogram (UH) approach [30] and its extension UH-proportion or simply UHp that we extend UH for the case where the total sample size of the released data is fixed and public. The descriptions of the UH and UHp approaches, the details of their implementation, and the results from the simulation study and the case study are presented in the supplementary materials. In summary, UHp delivers comparable performance to the flat sanitizer in bias and RMSE for most of the parameters in the simulation study but has slight under-coverage at $\epsilon =$ 1 and 0.5. UH performs the worst (largest bias, RMSE, and some notable under-coverage). In the case study, there is some discrepancy between the privacy-preserving point estimates vs the original for both UH and UHp. For UH, some CIs are noticeably wider than the original, mostly in the race/ethnicity groups that are relatively small in size.

## Privacy-preserving release of case location data

We examine a privacy-preserving approach to releasing location data based on GI. We focus on releasing cross-sectional location data at a given time point rather than travel trajectories [41], the latter being a topic for future research. Even though released data are cross-sectional, they can be released on a regular time basis, e.g., every day or every 3 days, allowing temporal examination of certain trends.

**Fig. 3:**
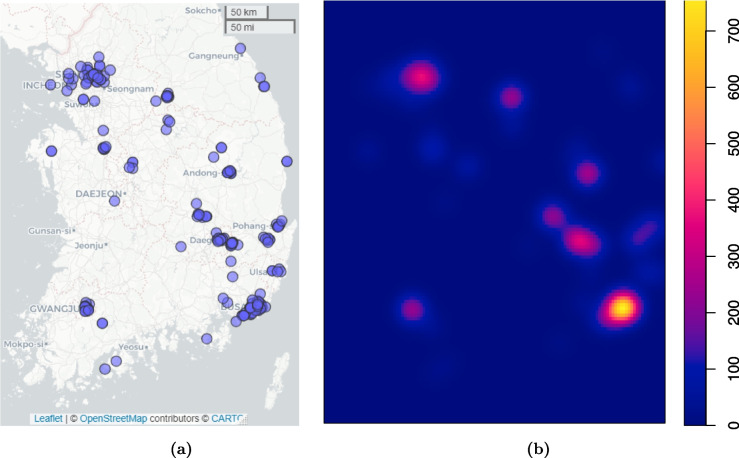
**a** Observed locations of 121 COVID-19 patients on Feb 20, 2020 in South Korea. **b** Hotspot heat map based on observed locations

An example of location data is given in Fig. 3, which shows the locations of 121 COVID-19 patients on Feb 20, 2020, in South Korea. The data can be found in the file “patientroute.csv” at https://www.heywhale.com/mw/dataset/5e797e9e98d4a8002d2c92d3/file. The number of locations per subject ranges from 1 to 11; about 50% (62 out of 121) has one location, 34.7% has 2 or 3 locations, and the rest 14% have $\ge 4$ locations (one person has 11 locations; all within the city of Gwangju). The timestamp information in hours, minutes, and dates is not available in the dataset.

### Method

The approach we propose for releasing privacy-preserving location information is *the doppelganger* [41], based on the GI concept. The main idea behind doppelganger, as suggested by the name, is to release $m\ge 1$ sanitized versions of the true location *P* via the planar Laplace mechanism so to satisfy GI guarantees. The privacy budget per location $\epsilon$ is split into *m* portion for $m\ge 2$, $\epsilon /m$ per release. Similar to case surveillance data, the main reason for releasing multiple perturbed locations ($m\ge 2$) is to provide a way to quantify sanitization uncertainty and draw statistical inferences using the MS approach.

To generate a sanitized location $(x^{*},y^{*})$ given the original location coordinates (*x*, *y*), we apply the planar Laplace mechanism in Eq. (3), with $\epsilon$ replaced by $\epsilon /m$. $\epsilon$ is the per-unit-distance privacy loss, where the unit distance is supplied by the data curator and can be any value deemed appropriate for the task at hand, such as 1 meter, 10 meters, 0.5 miles, etc (generally speaking, the choice depends on location type, area, among other considerations). In many cases of location sanitization, there is public knowledge of where the locations belong and how many cases there are. For example, in the South Korean data, all cases are on the land of South Korea, instead of from its neighboring nations such as Japan or China, or in the ocean. Therefore, one would expect sanitized locations to be in the land of South Korea as well, and post-processing bounding will be applied to the out-of-bound sanitized locations. Algorithm 2 summarizes the steps of the sanitization procedure.

**Figure:**
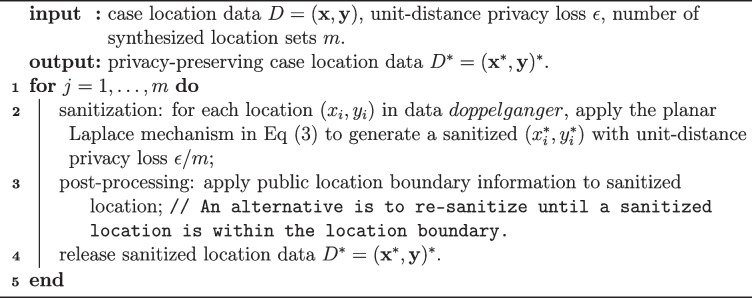
**Algorithm 2** Privacy-preserving release of case location data via geoindistinguishability

### Simulation study

To evaluate the statistical utility of sanitized locations via doppelganger, we conduct a simulation study. We simulated 1,000 sets of location data in a square area of $[0, 1]\times [0, 1]$ from an inhomogeneous Matérn cluster point process with the radius of the clusters at 0.03 and the non-stationary log-density $\log (\lambda (x,y;\varvec{\beta }))= \beta _0+\beta _1x+\beta _2y+\beta _3x^2+\beta _4y^2+\beta _5xy$, where *x* and *y* are coordinates and $\varvec{\beta }=(\beta _0,\ldots ,\beta _5)=(4.53, 3.30, 3.43, -0.27, 1.58, 2.24)$. The number of locations ranges from 769 to 1217 across the 1,000 repeats with an average of 970. In each simulated dataset, we sanitized each location with the planar Laplace mechanism in Eq. (3) at $\epsilon =5, 2, 1, 0.5$ per 0.01 unit and $m=3$. We assume $[0, 1]\times [0, 1]$ is public information and sanitized locations thus should fall within $[0, 1]\times [0, 1]$. On the other hand, the planar Laplace mechanism can generate an infinite *r* and any angle $\in [0,2\pi ]$. To honor the location boundaries, we set sanitized $x^{*}<0$ at 0 and at 1 if it is $>1$; similarly for sanitized $y^{*}$. We then fitted the inhomogeneous Matérn cluster point process model above and applied the inferential rule in Eq. (6) to obtain inference on $\varvec{\beta }$. The data simulation and analysis were conducted using R package spatstat.core [6].

The results are presented in Table 3. In summary, the inferences at $\epsilon =5$ and $\epsilon =2$ are comparable to the original – close-to-0 bias, similar RMSE as the original, nominal converge at $\epsilon =5$ and slight under-coverage at $\epsilon =2$. At $\epsilon =1$ and $\epsilon =0.5$, the bias is notable; the RMSE values are similar to the original at $\epsilon =1$, but much larger at $\epsilon =0.5$; the CP is around 83% to 85% at $\epsilon =1$ and ranges from 60% to 88% at $\epsilon =0.5$. The moderate to severe under-coverage is largely due to the bias in the $\varvec{\beta }$ estimates, which in turn may be attributed to the bounding applied to the sanitized locations. Bounding sanitized values can lead to biased inference [39].

**Table 3:** Privacy-preserving inferences of Matérn cluster point process model on simulated location data (1,000 repeats; $m=3$)

Metric	Parameter	Original	$\epsilon =5$	$\epsilon =2$	$\epsilon =1$	$\epsilon =0.5$
	$\beta _0$	-0.029	-0.022	0.016	0.142	0.571
	$\beta _1$	0.065	0.052	-0.022	-0.279	-1.180
bias	$\beta _2$	0.031	0.014	-0.074	-0.374	-1.389
	$\beta _3$	-0.085	-0.077	-0.028	0.154	0.801
	$\beta _4$	0.034	0.038	0.060	0.124	0.337
	$\beta _5$	-0.037	-0.024	0.048	0.303	1.160
	$\beta _0$	0.466	0.465	0.459	0.457	0.680
	$\beta _1$	1.234	1.232	1.211	1.189	1.549
RMSE	$\beta _2$	1.164	1.162	1.152	1.166	1.693
	$\beta _3$	1.006	1.003	0.986	0.958	1.159
	$\beta _4$	0.944	0.943	0.934	0.898	0.838
	$\beta _5$	0.985	0.982	0.972	0.989	1.431
	$\beta _0$	0.948	0.940	0.925	0.841	0.599
	$\beta _1$	0.938	0.932	0.914	0.845	0.719
CP	$\beta _2$	0.957	0.952	0.935	0.851	0.640
	$\beta _3$	0.938	0.929	0.909	0.842	0.769
	$\beta _4$	0.941	0.934	0.908	0.840	0.878
	$\beta _5$	0.947	0.939	0.916	0.827	0.638

### Application to South Korea case location data

We apply the doppelganger to the real South Korean case location dataset (Fig. 3(a)) to release privacy-preserving locations at $\epsilon =5, 2, 1, 0.1$ per 2 miles per individual. For an individual who has more than one location record, we further divided $\epsilon$ by the number of locations for that individual. That is, if an individual has *h* original location data points and we release *m* sanitized locations for each location at a privacy budget of $\epsilon /(mh)$. Similar to the simulation study, we honor the fact that all cases are in South Korea and bounded sanitized locations within a rectangular that approximates the shape of South Korea, in a similar fashion as done in the simulation study.

**Fig. 4:**
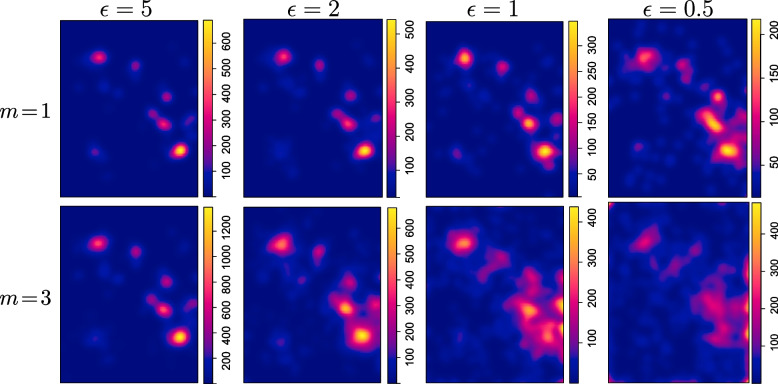
Privacy-preserving COVID-19 hotspot heat maps in South Korea on Feb 20, 2020

We used two analyses to check the utility of the sanitized locations: to generate hotspot heat maps and to fit a point process model. We set $m=3$ in both analyses but also examined $m=1$ in the former as it does not involve statistical inference. The privacy-preserving heat maps are displayed in Fig. 4 with the same smoothing bandwidth as in Fig. 3(b).

The privacy-preserving hotspot heat maps are very similar to the original heat map in Fig. 3(b) at $\epsilon \ge 1$ for both $m=1$ and $m=3$ and are a bit noisy at $\epsilon =0.5$ especially when $m=3$; but the major hotspots (the cities of Busan, Seoul, and Daegu) are preserved at $\epsilon =0.5$ for $m=1$. In summary, for the purposes of generating heat maps, $m=1$ is sufficient and each sanitized location is less noisy compared to using $m=3$, especially at small $\epsilon$.

We fitted an inhomogeneous Matérn cluster point process model with log-density $\log (\lambda (x,y;\varvec{\beta }))=\beta _0+\beta _1x+\beta _2y$ on the original data and the sanitized data. For this analysis, we randomly selected one location if an individual has multiple original location records, resulting in one original location per individual. We applied the inferential rule in Eqs. (5) and (6) to obtain the point estimates and 95% CIs for $(\beta _0,\beta _1,\beta _2)$.

The results are presented in Table 4. In general, the privacy-preserving inferences are similar to the original, especially for $\beta _1$ and $\beta _2$ that quantify the linear trends of COVID intensity along the *x* and *y* coordinates, respectively. In addition, the privacy-preserving point estimates are robust to $\epsilon \ge 1$ and some notable deviation from the original is only seen at $\epsilon =0.5$. A surprising observation is a shrinkage in the CIs as $\epsilon$ decreases for $\epsilon <5$, implying the inferences become more precise, at least for the range of the examined $\epsilon$ values, though the statistical insignificance remains unchanged across $\epsilon$. The shrinkage is counter-intuitive as one would expect the inferences to get less precise as the locations are perturbed more at smaller $\epsilon$. Indeed, as $\epsilon$ decreases, the sanitized locations are more scattered (Fig. 4), but the likelihood of a sanitized location being bounded at the boundary also increases, which may affect the within and between components of the total variance in Eq. (5). More research is needed to better understand how the variability is affected by the sanitization and the bounding constraint.

**Table 4:** Privacy-preserving Matérn cluster point process model parameter estimates based on sanitized locations in the South Korea location data ($m\!=\!3$)

	Estimate (95% CI)				
	Original	$\epsilon =5$	$\epsilon =2$	$\epsilon =1$	$\epsilon =0.5$
$\beta _0$	-64.2 (-153.5, 25.1)	-65.1 (-157.0, 26.9)	-63.0 (-147.0, 21.0)	-63.8 (-140.6, 13.0)	-57.5 (-129.8, 14.7)
$\beta _1$	0.51 (-0.17, 1.19)	0.52 (-0.18, 1.21)	0.50 (-0.14, 1.14)	0.50 (-0.08, 1.08)	0.44 (-0.10, 0.99)
$\beta _2$	0.03 (-0.50, 0.56)	0.03 (-0.52, 0.59)	0.03 (-0.48, 0.54)	0.05 (-0.42, 0.51)	0.07 (-0.39, 0.53)

### Summary

The doppelganger method releases location data with privacy guarantees. The simulation study and the case study suggest the method can preserve important statistical signals in the original data at a relatively low-level cost of privacy. The method would be particularly useful for protecting location privacy when sharing information at a local level or releasing hotspot maps on a relatively fine scale. The finer the scale is, the more sparse the data become, the higher the privacy risk for re-identification from releasing location data, and the greater the need for effective privacy protection approaches, but also the noisier released sanitized locations. As the scale gets coarser, say at the city, regional, state, or national levels, the information released by the doppelganger can be very similar to the original location information.

## Privacy-preserving sharing of contact tracing networks

CT data are often collected as needed, that is, when a person is diagnosed positive for COVID-19. In those cases, a CTN may only contain COVID-positive individuals and their close contacts. That said, CTNs can be constructed in different ways from CT data, and they can be complex and large as people are mobile and may show up in various places at different times. We focus on CTNs constructed for a pre-defined population during a pre-specified period of time (e.g., employees in an organization or students in a school in one day, 2 weeks, or 1 month, etc). For example, suppose the time period is one day, starting at noon on June 1 2020 ending at noon on the next day and the population is all students at a college. If a COVID-positive student named Tom was in a dining hall from noon to 1 pm on June 1, 2020, and had 2 close contacts, at the library from 1:30 pm to 5 pm and had 1 close contact, and in his dorm from 5 pm to noon next day and had 5 close contacts, then Tom and all his 8 close contacts are included in the CTN, along with 8 edges, representing the 8 close contacts. We consider the privacy-preserving release of CTNs with relational information only in this study; releasing CTNs with nodal attributes (such as demographic information or location information) with privacy guarantees is a topic for future research.

### Method

We examine a few approaches for releasing privacy-preserving CTNs and present one approach, DP-ERGM, in the main text and include the other two in the supplementary materials. DP-ERGM stands for Differentially Private network synthesis via Exponential Random Graph Model [42]. The DP-ERGM procedure can be regarded as an application of the model-based differentially private synthesis (MODIPS) approach [40] to graph data with ERGM as the synthesis model. ERGMs are a family of popular statistical models for analyzing network data [51, 53]. Denote by $\mathcal {E}$ the adjacency matrix in a network ($e_{ij}=1$ if an edge exists between node *i* and node *j*, $e_{ij}=0$ otherwise). ERGMs model the conditional distribution of $\textbf{e}$ as

7 $$\begin{aligned} p(\mathcal {E} |\varvec{\theta }) = \frac{\textrm{exp} \left\{ \varvec{\theta }^{T} \textbf{S}(\mathcal {E}) \right\} }{K(\varvec{\theta })} \text {, where}\ K(\varvec{\theta })= \sum _{\mathcal {E}'} \textrm{exp} \left\{ \varvec{\theta }^{T} \textbf{S}\mathcal ({E}') \right\} , \end{aligned}$$

where $\textbf{S}(\mathcal {E})$ is the summary statistics that characterize the network structure such as the number of edges, degree distribution, edge-wise shared partnership, etc. $K(\varvec{\theta })$ is the normalizing constant summed over all possible adjacency matrix $\textbf{e}'$ and is often analytically intractable unless in small networks. Inference of $\varvec{\theta }$ is often based on approaches with approximate $K(\varvec{\theta })$, such as the Monte Carlo maximum likelihood estimation [27, 32]. Equation 7 is a simplified ERGM as we deal with CTN without nodal attributes in this study. In general, $\textbf{S}$ may contain statistics not only constructed from $\textbf{e}$ but also nodal statistics for networks with nodal attributes.

The steps of a general DP-ERGM procedure are presented in Algorithm 3. Regarding the ERGM on which the likelihood is based, it may be specified prior to the access to $\mathcal {E}$ or chosen using a privacy-preserving procedure given by $\mathcal {E}$, costing a portion of the total privacy budget $\epsilon$. Regarding posterior sampling with a pre-set privacy loss, readers may refer to [24, 40] for some of the available approaches; other options are through differentially private MCMC sampling, such as Heikkilä et al. [31], Li et al. [36], Seita et al. [52] is naturally differentially private. Balle and Wang [7], Yıldırım and Ermiş [62] show that the penalty method for Metropolis-Hastings (MH) algorithms Wang et al. [55]

**Figure:**
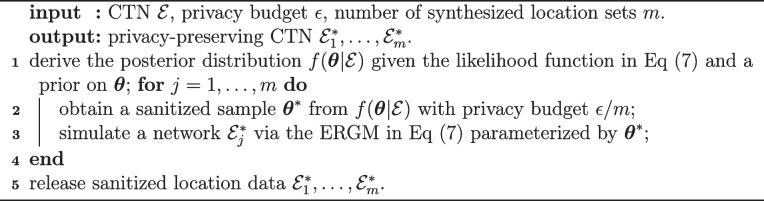
**Algorithm 3** Privacy-preserving release of CTN via DP-ERGM

In addition to DP-ERGM, we also examined a random response (RR) mechanism for perturbing edge information with DP guarantees [34] and a debiased version of the RR mechanism [42]. Both procedures perform significantly worse than the DP-ERGM procedure in the utility analysis performed in Section Simulation Study unless the privacy loss is high ($\epsilon >5$). The details on RR and RR-debias can be found in the supplementary materials.

### Simulation study

To evaluate the statistical utility of sanitized CTNs, we conduct a simulation study. We simulated 500 sets of networks from an ERGM model with a single covariate *s* (edge count). In each simulated network, there are 100 nodes. The networks were simulated to mimic real-life CTN (a CT dataset collected at the University of Notre Dame, USA, during the pandemic) in the degree distribution per individual. The real data are not shareable due to privacy and IRB reasons.

The ERGM used in the DP-ERGM procedure contains edge count as a single covariate. We applied an approach in Liu [40] to draw a privacy-preserving posterior sample on $\theta$ and also sanitized the edge count via the Laplace mechanism, which has a sensitivity of 1 (flipping a relation between two nodes changes the edge count in a network by at most 1). We equally split the total privacy budget $\epsilon$ between drawing a posterior sample of $\theta$ and sanitizing the edge count given a network. Given the privacy-preserving sample of $\theta$ and the sanitized edge count, we generated a privacy-preserving CTN under the constraint that its edge count equals to the sanitized edge count. We examine $\epsilon \!=5, 2, 1, 0.5$. The ERGM model fitting and network simulation were completed using R package statnet [29]. We conduct two utility analyses. In the first analysis, we examine the preservation of qualitative information and descriptive statistics in sanitized CTNs; in the second analysis, we run the ERGM on sanitized networks to examine the inference on the model parameter. *m* is set at 1 and 3, respectively, in these two analyses.

For the first utility analysis, we calculate some common network summary statistics, including edge counts, triangle counts, degree distribution (DD), edgewise shared partners distribution (ESPD), and two-node centrality measures in a sanitized network. Edge and triangle counts are the numbers of edges and triangles in a network. The DD in a network with *n* nodes consists of $d_k$ for $k\!=\!0,\ldots ,n\!-\!1$, where $d_k$ is the number of nodes that share an edge with exactly *k* other nodes. The ESPD consists of $\textrm{esp}_k/\text {edge count}$ for $k\!=\!1,\ldots ,\le n(n-1)/2$, where $\textrm{esp}_k$ is the number of edges whose two nodes are both connected with exactly *k* other nodes than themselves. The betweenness centrality measures the centrality of a node in a graph and is defined for node *i* as the proportion of the shortest paths that connect nodes *j* and $j'$ while passing through node *i* ($j\ne j'\ne i)$ among all shortest paths that connect nodes *j* and $j'$. There are multiple definitions of closeness centrality and we use $\left( \frac{A_i}{n-1}\right) ^2/C_i$, where $A_i$ is the number of reachable nodes from node *i*, and $C_i$ is the sum of distances from node *i* to all reachable nodes. If no nodes are connected with node *i*, its closeness centrality is 0.

**Fig. 5:**
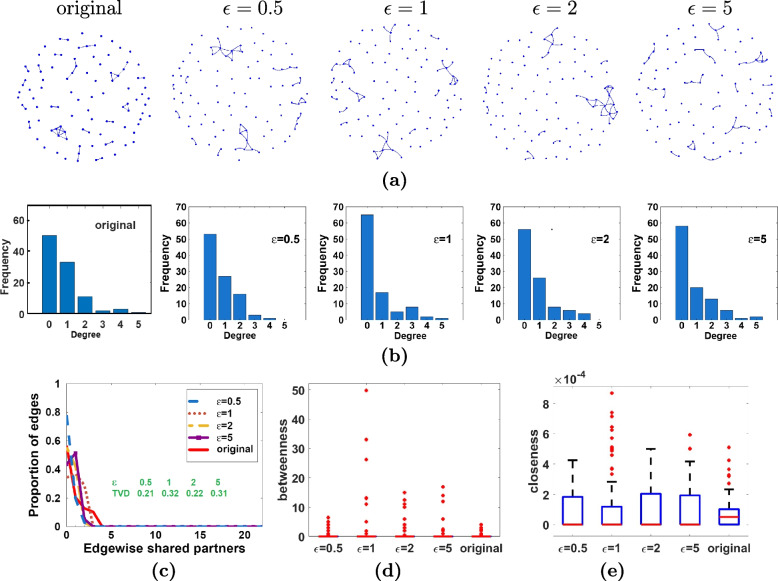
Comparison between original and sanitized CTNs on various network structural statistics. **a** examples of sanitized CTNs. **b** degree distribution. **c** Edgewise shared partner distribution (ESPD). **d** betweenness centrality. **e** closeness centrality

The visualization of a single sanitized CTN from one of the 500 repeats is presented in Fig. 5(a) and provides a big-picture comparison between the sanitized vs the original networks in terms of density, clustering, etc. In summary, the density of the sanitized CTNs via DP-ERGM is similar to the original CTN at all the examined $\epsilon$ values. Note the nodes in the sanitized networks do not match the nodes in the original CTN as DP-ERGM samples a whole new surrogate network from a differentially private ERGM model for release. The edge and triangle counts of the original networks are 39 and 10, respectively. The average (standard deviation) edge counts over 100 sanitized CTNs are 38 (6.4), 39 (3.1), 39 (1.4), and 39 (0.7) at $\epsilon =0.5, 1, 2,$ and 5, respectively; the average (standard deviation) triangle counts over 100 sanitized CTNs are 13 (9.2), 12 (7.4), 11 (6.8), and 11 (7.1) at $\epsilon =0.5, 1, 2,$ and 5, respectively. These numbers are consistent with the observations in Fig. 5(a). Figures 5(b) and 5(c) depict the DD and ESPD of the sanitized CTN. In the latter, we also calculate the total variance distance (TVD) in ESPD between the sanitized and original CTNs, which are presented in Fig. 5(c). Figures 5(d) and 5(e) show the box plots of the betweenness centrality and closeness centrality of the 100 nodes in the original and sanitized CTNs. Though there is some deviation in the DD, ESPD, and the distributions of the centrality measures in the sanitized CTNs from the original, the deviation is rather mild. In addition, the statistics are relatively stable across $\epsilon$.

For the second utility analysis, we fitted the ERGM on the sanitized CTNs to obtain privacy-preserving inference on $\theta$, the coefficient associated with edge count in ERGM, via the inferential rule in Eqs. (5) and (6). The results are presented in Table 5. In summary, the results are acceptable for the ERGM analysis at all examined $\epsilon$ (especially for CP).

**Table 5:** Inference of ERGM parameter based on sanitized CTNs ($m=3$; 500 repeats)

	Original	$\epsilon =5$	$\epsilon =2$	$\epsilon =1$	$\epsilon =0.5$
bias	-0.021	-0.021	-0.026	-0.031	-0.051
RMSE	0.171	0.172	0.174	0.187	0.260
CP	0.942	0.954	0.954	0.952	0.944

### Summary

The simulation study suggests that the DP-ERGM approach can produce privacy-preserving CTNs that are structurally similar to original CTNs by various statistical measures. In addition, the utility of sanitized CTNs is relatively insensitive to $\epsilon$ for the examined range of [0.5, 5], implying that a small $\epsilon$ can be used to provide strong privacy guarantees without sacrificing much of the utility. The sanitized CTNs can be shared with researchers who are interested in learning more about CTNs during the pandemic, without compromising individual privacy at a pre-specified privacy cost.

## Conclusions

We use three common data types – surveillance case numbers, case location information, and contact tracing networks – collected during the COVID-19 pandemic to demonstrate the release and sharing of privacy-preserving data. In each data case, we apply randomized mechanisms with formal privacy guarantees to sanitize and release information aiming at the preservation of statistical utility and aggregate information that can be used to infer underlying population parameters, as shown in the simulation studies and real-life applications. The approaches do not target learning individual-level information, which not only conflicts with the goal of privacy protection but is also unnecessary for the purposes of mining and understanding population-level information.

DP and its various extensions are state-of-the-art concepts in privacy research and are quickly adopted in practice. Some of the methods we have demonstrated in the study are basic and have been routinely applied for privacy protection, such as the flat sanitizer; and some are recently proposed, such as DP-ERGM. For all the data types and examples examined in this study, synthetic data are generated and released at a pre-specified privacy budget and users may perform their own analysis on the synthetic data without having to worry about additional privacy loss. Our simulation studies suggest that different DP procedures for a given statistical analysis procedure may lead to different utilities of sanitized information and also vary in the easiness of implementation, an observation well documented in the literature and also one of the reasons why new DP methods are constantly proposed to improve on the existing methods with either better utility or more straightforward implementation. In addition, absolute privacy protection for individuals in a dataset only exists on paper unless the released information is completely random or independent of the dataset. In reality, there is always some loss of privacy when releasing new and useful information; the choice of a proper privacy loss is a key step when implementing DP procedures.

We hope our study and the examples shed light on the privacy-preserving sharing of COVID-19 data to help promote and encourage more data sharing for research use. For future work on this topic, we will continue to develop methods to deal with more complicated COVID-19 data-sharing situations, such as releasing travel trajectories of COVID-19 patients, longitudinal data, and dynamic CTNs, CTNs with nodal attributes, among others.

## Supplementary Information

**Additional file 1.**

## Data Availability

The data that support the findings of this study are openly available at https://www.cdc.gov/nchs/nvss/vsrr/covid19/health_disparities.htm (Table 2; May 24, 2022) and https://www.heywhale.com/mw/dataset/5e797e9e98d4a8002d2c92d3/file (file “patientroute.csv”).
