# Supplementary material for: Some examples of privacy-preserving sharing of COVID-19 pandemic data with statistical utility evaluation
Source: BMC Med Res Methodol. 2023 May 19;23:120. doi: 10.1186/s12874-023-01927-3 (PMC10198035; doi:10.1186/s12874-023-01927-3)
Supplement: Supplementary file 1 — Additional file 1. [file 12874_2023_1927_MOESM1_ESM.pdf]

Supplementary Materials to "*Some Examples of Privacy-preserving Sharing of COVID-19 Pandemic Data with Statistical Utility Evaluation*"

Fang Liu, Dong Wang, Tian Yan

UH and UHp for Surveillance Case Number Release

The UH approach forms a hierarchical tree among the data attributes and injects noise to each node count in each layer of the tree, explores equality constraints between each parent node and its children nodes in the tree to improve the accuracy of the sanitized count of the parent nodes (low-order marginals) and release the final corrected counts from the whole tree. Figure S1 displays a 4-layer hierarchical tree formed in the UH approach on a data set with 3 variables (age group, minority/majority, sex). We refer to the node at the top of the tree as the root (layer 1) and those at the bottom as the leaf nodes (layer 4). The age nodes at layer 3 are parents to the race/ethnicity nodes in layer 3, which are the parent nodes to the sex nodes in layer 4. There is no particular ordering among the three attributes in the example in Figure S1. We can place the attributes in the middle layers of the trees that would enjoy a lower mean squared error (MSE) (MSE for a sanitized count $\tilde{x}$ is $\mathbb{E}_{\mathcal{M}}(\tilde{x}-x)^2$, where x is the original count and the expectation is taken over the distribution of the randomized algorithm). in their marginal sanitized counts relative to their original counts, compared to the MSE resulting from a simple sum of the directly sanitized counts of the most granular cells as done in the flat sanitizer.

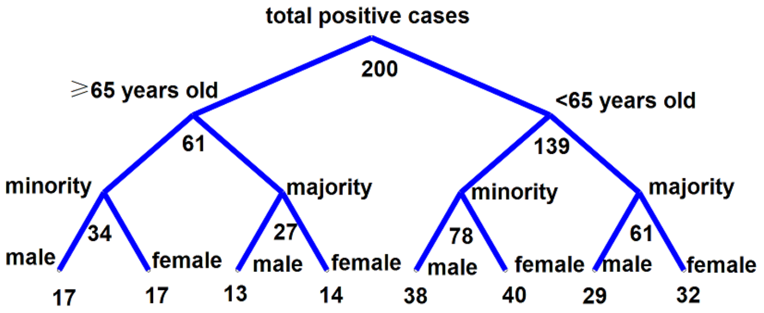

Figure S1: A count hierarchical tree with three binary attributes

The UH procedure is implemented in 3 steps. First, since each layer is sanitized, the total budget ϵ should be split among the layers following the sequential composition principle in DP [2]. For illustration purposes, we assume each layer receives $1/l$ of the total ϵ, where l is the height of the tree (other privacy allocation schemes across the layers can also be used). and $l = 4$ in Example 1. The count $h[v]$ in each node v in the tree is sanitized via the Laplace mechanism $\text{Lap}(0, l\epsilon^{-1})$; that is, $\widetilde{h}[v] = h[v] + e$, where $e \sim \text{Lap}(l\epsilon^{-1})$, where $\widetilde{h}[v]$ is the sanitized count. In step 2, intermediate node count $z[v]$ for each node v is obtained via Eq (S1),

$$z[v] = \begin{cases} \widetilde{h}[v], \text{ if } v \text{ is the leaf node} \\ \frac{k^l-k^{l-1}}{k^l-1}\widetilde{h}[v] + \frac{k^{l-1}-1}{k^l-1}\sum_{u\in succ(v)} z[u], \text{ o.w.} \end{cases}, \tag{S1}$$

where succ(v) denotes the set of children nodes to parent node v and k is the number of children per parent node, which is assumed to be the same for each parent ($k = 2$ in example 1). The reason behind Eq (S1) is that for the nodes not from the bottom layer (the non-leaf nodes), a sanitized count comes from two sources (the node being sanitized, and the summation from its children nodes) so Eq (S1) calculates a weighted average of the two. Obviously, $z[v]$ may no longer be equal to the sum of the node counts of its children nodes, violating the equality constraints in contingency tables. This inconsistency is corrected via Eq (S2), yielding the final sanitized count $h^*[v]$

$$h^*[v]=\begin{cases} z[v] \text{ if } v \text{ is the root node} \\ z[v] + k^{-1}\left(h^*[u] - \sum_{w\in succ(u)} z[w]\right), \text{ o.w.} \end{cases}, \tag{S2}$$

where u is the parent mode to node v, succ(u) contains the children nodes to parent node u, and $h[u] - \sum_{w\in succ(u)} z[w]$ is the correction term to ensure the equality constraint holds for each parent node in the tree.

We extend the UH approach to sanitizing a proportion tree (Figure S1) in place of a count tree and name it the UHp approach ("p" in the name "UHp" stands for "porportion"), in cases where the total sample size n is public information and can be released directly, or when it is desirable not to alter n from a statistical inferential perspective as n is critical for inferences such as inferential efficiency.

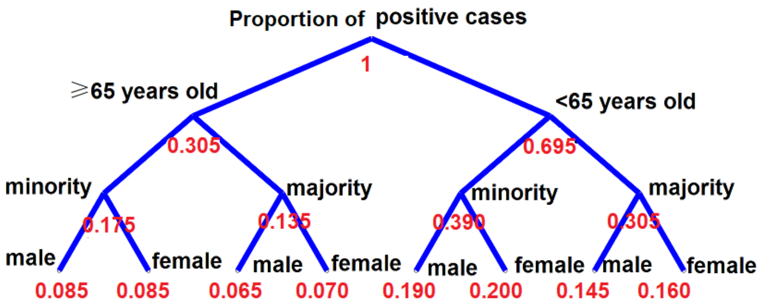

Figure S2: A proportion hierarchical tree with three binary attributes

The sanitization process for UHp is similar to UH with a few modifications. First, given the proportion at the top layer is always 1, there is no need to sanitize the node and the total ϵ is only needed to split into $l - 1$ layers. Second, the Laplace distribution from which the noise is drawn becomes Laplace$(0, (l-1)\epsilon^{-1}/n)$ as the global sensitivity for proportion is $1/n$. Third, after obtaining $\widetilde{h}[v] = h[v] + e$, where $e \sim$ Lap $(l - 1)\epsilon^{-1}/n)$ for all the non-root node proportions, we normalize the proportions in layer 2 as in $\widetilde{h}[v^{(2)}] = \widetilde{h}[v^{(2)}]/\sum_u \widetilde{h}[u]$, where u refers to all the nodes in layer 2, so that the layer-2 proportions sum up to 1, honoring the constraint of $\tilde{h}[v] = h[v] = 1$ for the root node. The steps in Eqs (S1) and (S2) after the normalization step remain the same as in the UH approach. After the sanitized proportions are obtained, the corresponding counts can be obtained by multiplying the proportions with the total n.

Similar to the flat sanitizer, the sanitized counts or proportions in the UH and the UHp approaches can be negative as the support of the Laplace distribution is $\mathbb{R}$. In addition, the

sanitized proportions may be > 1. We applied the same methods as used for the flat sanitizer to deal with negative counts and in the case of a fixed upper bound such as the proportions adding up to 1 and when the total count is fixed.

Simulation study and CDC death count application for UH and UHp

In the simulation study, for both UH and UHp, the tree height is $l = 4$ as there are 3 attributes – X_1 is layer 2, X_2 in layer 3, and X_3 in layer 4 – and $k = 2$ as all attributes are binary. The simulation results are presented in Figure S3, together with the flat Laplace sanitizer and the original results for comparison.

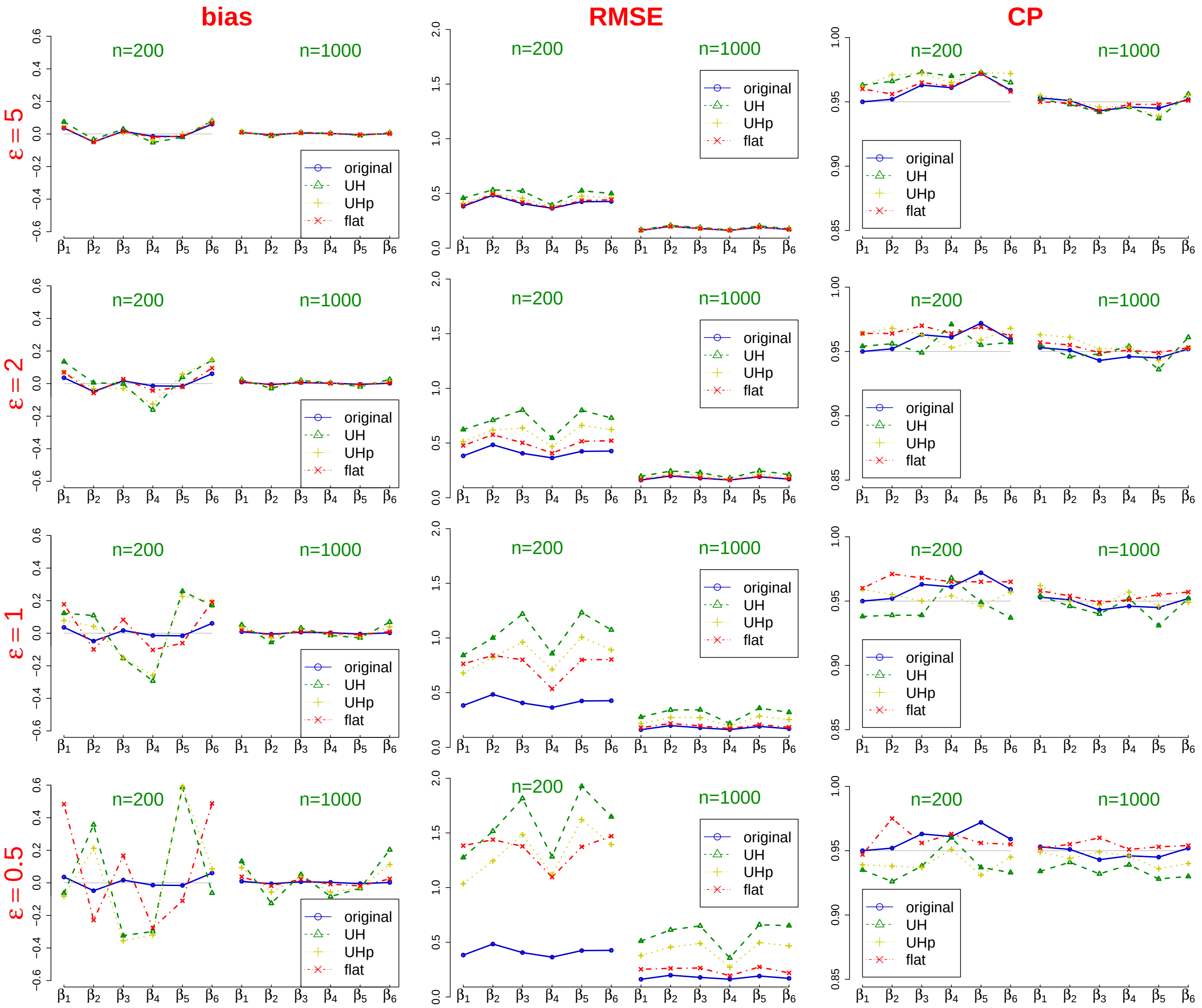

Figure S3: Privacy-preserving inference of the log-linear model based on sanitized counts by different methods in the simulation study ($m = 3$; 500 repeats)

For the application to the CDC COVID-19 death count data, $l = 3$ and $k = 7$ in the hierarchical tree for both UH and UHp. We placed age in layer 2 and race/ethnicity in layer 3 and don't expect the ordering would affect the results of the analysis we conducted in a statistically meaningful way. The results are presented in Figure S4, together with the flat Laplace sanitizer and the original results for comparison.

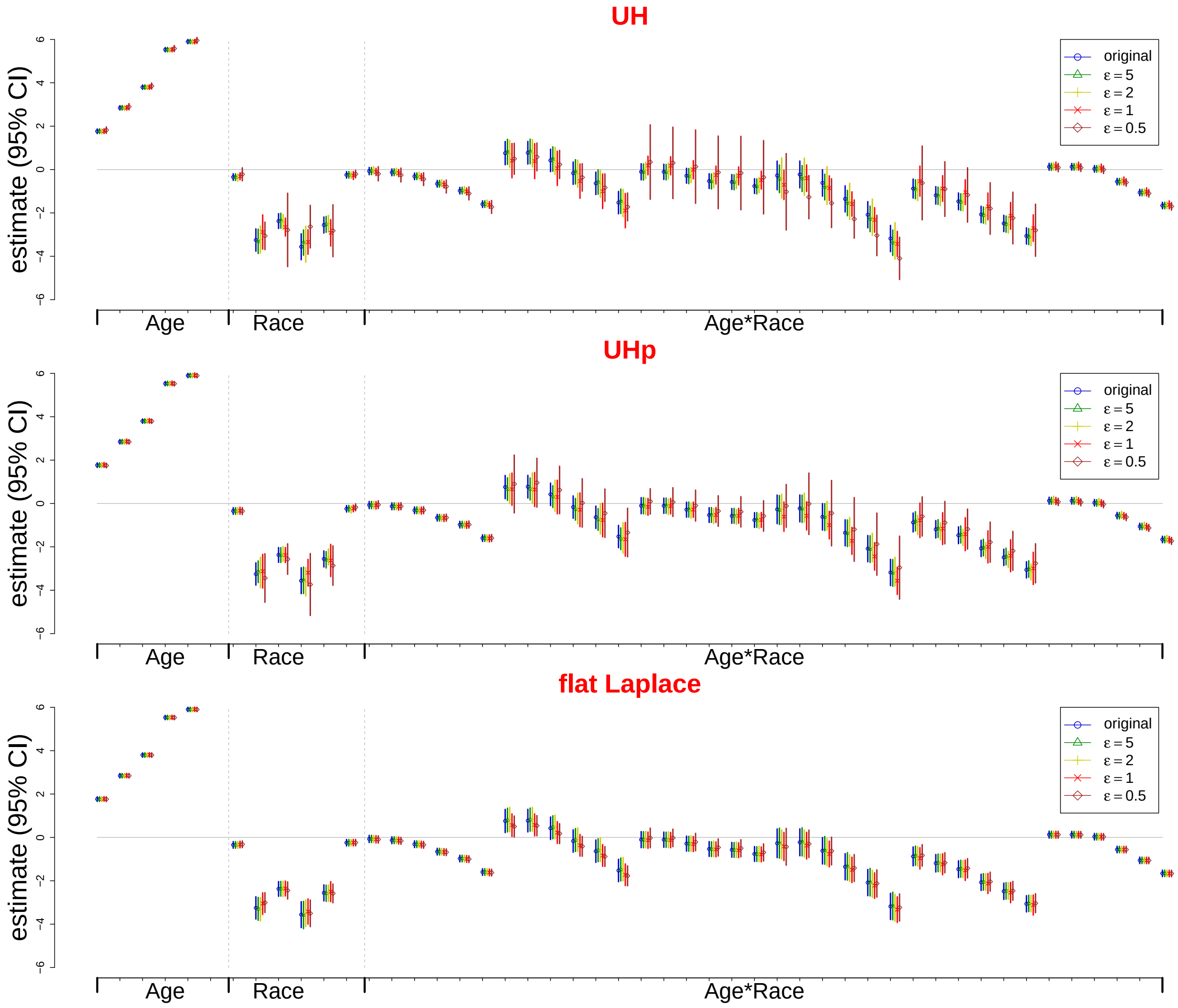

Figure S4: Privacy-Preserving results from the Log-linear model fitted on the CDC COVID-19 death data

Examples of sanitized US COVID-19 death counts by the Flat Laplace sanitizer ($m = 3$ and $\epsilon = 0.5$)

Table S1: Three sets of sanitized U.S. COVID-19 death counts by age group and race/ethnicity on May 24, 2022 ($m = 3; \epsilon = 0.5$) by the flat Laplace sanitizer

Age (ys)	Race/Ethnicity							
group	NH White	NH Black	NH AIAN	NH Asian	NH NHPI	NH Mix	Hispanic	Total
<17	385	273	19	38	14	25	302	1056
18-29	2265	1480	183	181	33	78	2018	6238
30-39	6665	4140	570	561	151	160	5916	18162
40-49	17277	8939	1024	1200	267	313	13979	43000
50-64	97407	35752	3196	5310	703	955	43655	186979
65-74	141417	37760	2913	7431	498	914	38416	229350
>75	380635	54588	3210	16515	447	1383	56700	513478
Total	646051	142933	11115	31236	2114	3827	160986	998262
Age (ys)	Race/Ethnicity							
group	NH White	NH Black	NH AIAN	NH Asian	NH NHPI	NH Mix	Hispanic	Total
<17	387	289	19	34	11	35	304	1079
18-29	2273	1492	183	197	54	86	2014	6299
30-39	6660	4124	568	571	147	157	5917	18145
40-49	17269	8930	1029	1216	279	314	13972	43010
50-64	97386	35756	3200	5315	723	953	43647	186982
65-74	141407	37753	2890	7426	513	918	38420	229327
>75	380591	54568	3205	16518	450	1382	56706	513420
Total	645974	142912	11095	31278	2177	3846	160980	998262
Age (ys)	Race/Ethnicity							
group	NH White	NH Black	NH AIAN	NH Asian	NH NHPI	NH Mix	Hispanic	Total
<17	392	284	19	29	10	29	309	1072
18-29	2236	1507	189	196	48	62	2007	6243
30-39	6659	4146	563	562	150	153	5903	18135
40-49	17260	8933	1002	1208	287	316	13983	42987
50-64	97418	35743	3198	5312	719	964	43674	187027
65-74	141398	37767	2897	7437	516	903	38420	229339
>75	380604	54573	3192	16513	460	1391	56724	513458
Total	645966	142953	11060	31255	2190	3818	161020	998262

Race/ethnicity = 'unknown' is not included in the table.
NH = Non-Hispanic; AIAN = American Indian or Alaska Native; NHPI = Native Hawaiian or Other Pacific Islander; "Mix" means "more than one race"

The RR mechanism and the RR-debiased procedure

The RR mechanism for sanitizing edges in a network works as follows. Let p_{ij} denote the probability the original edge $e_{ij} = 1$ is retained and q_{ij} be the probability that $e_{ij} = 0$ is retained after sanitization for nodes $i \neq j = 1, \ldots, n$. To satisfy ϵ_{ij}-DP (ϵ_{ij} edge DP precisely speaking; see Liu et al. [1] for details) in the sanitization of the relational information $e_{ij} = 1$, one may set $p_{ij} = q_{ij} = e^{\epsilon_{ij}}/(1+e^{\epsilon_{ij}})$. When there is no particular reasons for using different ϵ_{ij} for different pairs of nodes, one may set $\epsilon_{ij} \equiv \epsilon$ and the probability of edge flipping in the network is

$$p_{ij} = q_{ij} \equiv 1/(1 + e^{\epsilon}). \tag{S3}$$

If all edges are mutually independent, the total cost for sanitizing the whole network is also ϵ per the parallel composition principle.

Liu et al. [1] employs a debiasing approach as an attempt to remove bias in sanitized networks via the RR mechanism (with edges e^*_{ij}) by synthesizing new networks with edges $\tilde{e}^*_{ij}$ given an RR-sanitized network. Specifically,

$$\tilde{e}^*_{ij}|e^*_{ij} = 1 \sim \text{Bern}(p_1), \text{ where } p_1 = \frac{(p + q - 1)q}{(2q - 1)p}, \tag{S4}$$

$$\tilde{e}^*_{ij}|e^*_{ij} \sim \text{Bern}(p_0), \text{ where } p_0 = \frac{q(p + q - 1)}{(1 - p)(2q - 1)}, \tag{S5}$$

where $q = e^{\epsilon}/(1 + e^{\epsilon})$ is the probability of retaining an original edge by RR and p is the proportion of all $e^*_{ij} = 1$ in row i of the adjacency matrix of the synthetic network generated by RR (without the diagonal element), and. Synthetic networks via RR-debiased can be summarized and analyzed in the same way as the original network including descriptive statistics, visualization, and inference. For inference, there is no need to explicitly model the RR mechanism or the subsequent debiasing/sanitization process if $m > 1$ sets of synthetic networks are released. The debiasing procedure does not use the information from the original network and thus maintains the privacy guarantees, but at the cost of introducing another layer of variability. The debiased sanitized network is made of edges Y^* drawn from two Bernoulli distributions, depending on whether the synthetic edge Y' from the DWRR is 1 or 0.

Simulation Study on RR and RR-debiased

For RR, the probability of flipping an edge per Eq (S3) is $(1 + e^5)^{-1} = 0.7\%, (1 + e^2)^{-1} = 11.9\%, (1+e)^{-1} = 26.9\%$ and $(1+e^{0.5})^{-1} = 37.5\%$ at $\epsilon = 5, 2, 1, 0.5$, respectively. Though the probability of retaining the original relation between nodes i and j is very low at $\epsilon = 5$, the number of edges is expected to double $(39e^5/(1 + e^5) + (4950 - 39)e^5/(1 + e^5) = 71.6$ where 39 is the edge count in the original network).

The sanitized CTNs via RR and RR-debiased are presented in Figure S5 with the original CTN presented for comparison. Table S2 presents the number of edges and the number of triangles of the sanitized CTNs via RR and RR-debias. Figure S6 shows the DD, which is the distribution of close contacts of an individual in a CTN via RR and RR-debias, and Figures S7 depicts the ESPD of the sanitized CTNs with the TVD in DD between the sanitized and

original CTNs. Figure S8 shows the box plots of the betweenness centrality and closeness centrality of the 100 nodes in the sanitized CTNs via RR and RR-debias vs the original.

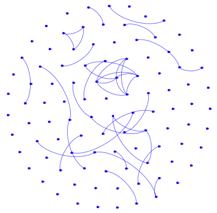

(a) original

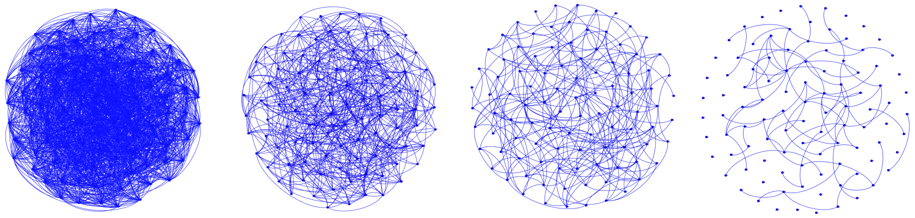

(c) RR

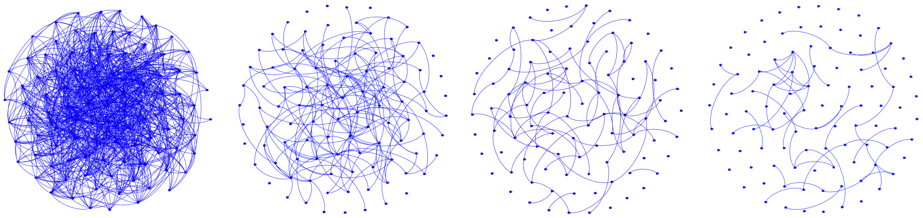

(d) RR-debiased

Figure S5: Examples of differentially privately sanitized CTNs via RR and RR-debiased

Table S2: Average (SD) number of edges and number of triangles over 100 repeats

	RR		RR-debiased	
ϵ	number of edges	number of triangle	number of edges	number of triangle
0.5	1876 (37.4)	8802 (528.3)	844 (82.4)	1258 (356.4)
2	619 (20.9)	320 (38.1)	182 (21.9)	14 (6.1)
5	72 (5.2)	10 (0.9)	48 (6.2)	6 (2.5)
8	40 (1.3)	10 (0.3)	40 (1.4)	9 (0.7)
10	39 (0.5)	10 (0.1)	39 (0.4)	10 (0)

original: number of edges = 39; number of triangle = 10. .

The results on the privacy-preserving inference of the ERGM based on the sanitized CTNs via RR and RR-debias are presented in Table S3. We present the results for $\epsilon = 5, 15, 18, 24$; the results at $\epsilon < 5$ are even worse.

Table S3: Privacy-preserving Inference of β in the ERGM model based sanitized CTNs via RR and RR-debiased ($m = 3$; 500 repeats)

method	metric	$\epsilon = 5$	$\epsilon = 15$	$\epsilon = 18$	$\epsilon = 24$
RR	bias	3.260	0.627	0.269	0.023
	RMSE	3.260	0.635	0.299	0.165
	CP	0	0.002	0.366	0.832
RR-debiased	bias	1.500	0.305	0.147	0.008
	RMSE	1.501	0.330	0.204	0.165
	CP	0	0.366	0.704	0.830

Original data: bias = -0.021, RMSE = 0.171, and CP = 0.942.

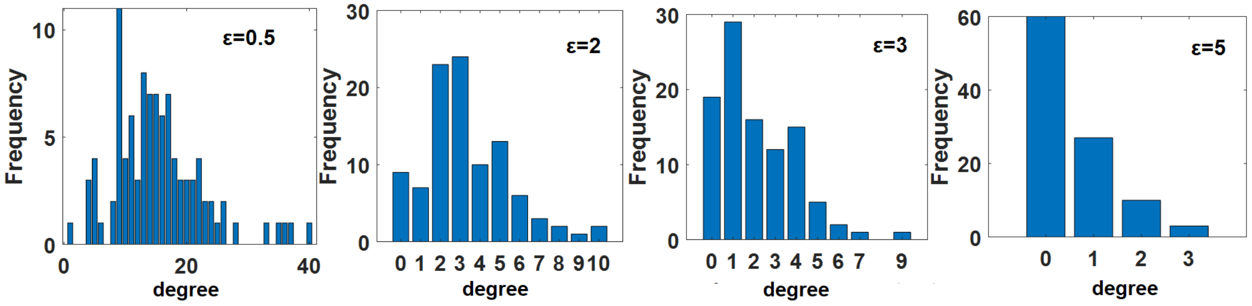

(a) RR

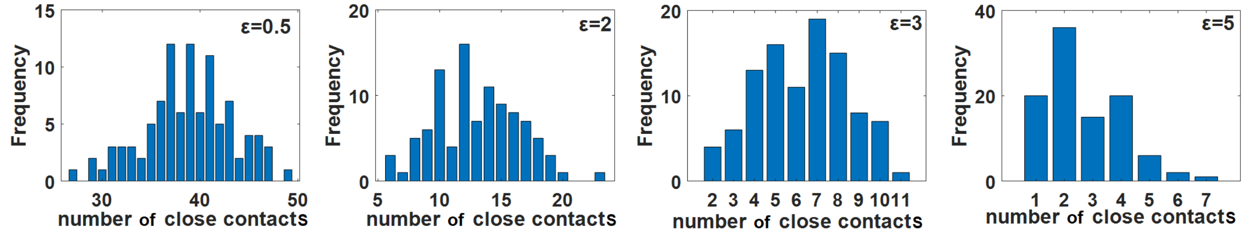

(b) RR-debiased

Figure S6: Degree distribution in the original and sanitized CTNs via RR and RR-debias

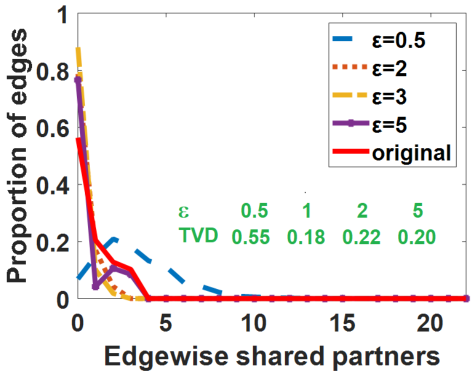

(a) RR

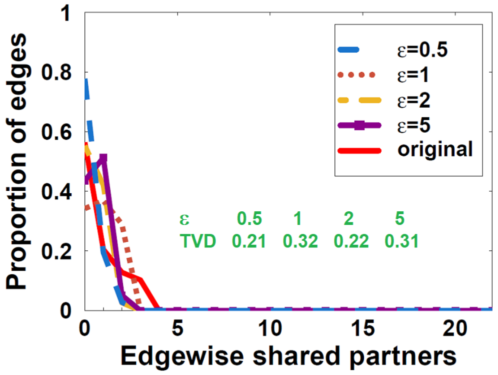

(b) RR-debiased

Figure S7: Edgewise shared partner distribution in sanitized CTNs via RR and RR-debias

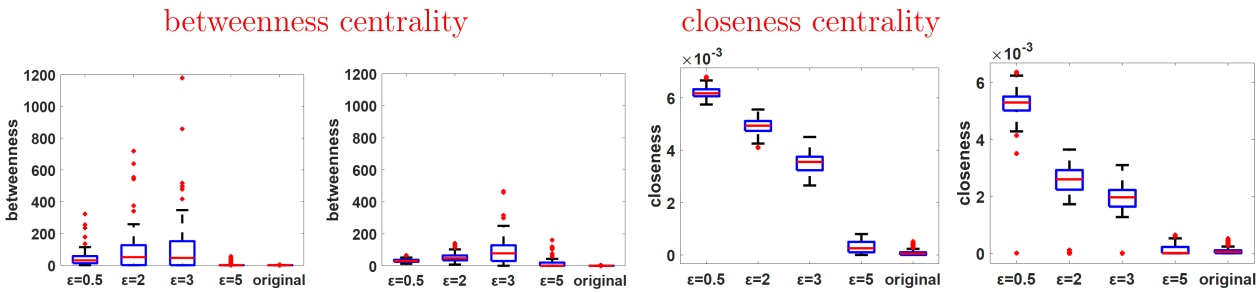

RR RR-debiased RR RR-debiased

Figure S8: Box plots of betweeness centrality and closeness centrality of 100 nodes in original and sanitized CTNs via RR and RR-debias

References

[1] Fang Liu, Evercita C. Eugenio, Ick Hoon Jin, and Claire Bowen. Differentially private synthesis and sharing of network data via bayesian exponential random graph models. *Journal of Survey Statistics and Methodology*, 10:2, 2022.

[2] Frank McSherry and Kunal Talwar. Mechanism design via differential privacy. In *48th Annual IEEE Symposium on Foundations of Computer Science (FOCS'07)*, pages 94–103, 2007.
